# Comprehensive real-time metabolic profiling of peripheral blood mononuclear cells reveals important methodological considerations for immunometabolism research

**DOI:** 10.3389/fimmu.2025.1676550

**Published:** 2025-10-29

**Authors:** Michael Macleod, Fendi Pradana, Alex J. Wadley, Jonathan Barlow

**Affiliations:** ^1^ Department of Inflammation and Ageing, Medical Research Council (MRC)-Versus Arthritis Centre for Musculoskeletal Ageing Research, College of Medicine and Health, University of Birmingham, Birmingham, United Kingdom; ^2^ School of Sport, Exercise, and Rehabilitation Sciences, University of Birmingham, Birmingham, United Kingdom; ^3^ Nutrition Study Program, Faculty of Public Health, Tadulako University, Palu, Indonesia; ^4^ Cellular Health and Metabolism Facility, College of Life and Environmental Sciences, University of Birmingham, Birmingham, United Kingdom

**Keywords:** PBMCs, bioenergetics, extracellular flux analysis, mitochondria, glycolysis, metabolic profiling, blood processing time

## Abstract

**Background/Introduction:**

The utility of measuring real-time cellular bioenergetics of peripheral blood mononuclear cells (PBMCs) as biomarkers in disease monitoring, such as the bioenergetic health index, is of emerging interest. However, various experimental factors can impact the accuracy and reproducibility of these measurements.Methods: PBMC bioenergetics were probed in real-time using extracellular flux analysis to identify optimal seeding density and injection protocol. Using a modified protocol, we assessed the extent to which blood processing time and isolation method (SepMate™ vs. EasySep™ Direct) influence PBMC bioenergetics under basal and stimulated conditions. Advanced metabolic control analysis including mitochondrial and glycolytic ATP supply flux, respiratory control ratio, bioenergetic health index, and mitochondrial toxicity index were used to identify and quantify PBMC bioenergetics.

**Results:**

Measures of metabolic profiling such as mitochondrial respiration, glycolytic activity, ATP supply flux, and respiratory control ratio were significantly diminished in PBMCs due to blood processing delay (48–72 hours) and were influenced by isolation method. Extended blood processing time significantly lowered T cell activation capacity in PBMCs, evidenced by decreased responses of mitochondrial and glycolytic ATP supply to CD3/CD28 activation.

**Discussion/Conclusion:**

This study demonstrates that key experimental variables including blood processing time and isolation method critically affect the reliability and biological relevance of PBMC metabolic assessments, highlighting the importance of protocol standardisation for accurate bioenergetic biomarker measurements.

## 1 Introduction

Immunometabolism is an emerging field originating from the intrinsic overlap between immunology and cellular metabolism. This has been facilitated by modern technological advances and recent acknowledgement that metabolic pathways are implicated in functions beyond cellular energy provision, for example, the modulation of intercellular signalling pathways and the post-translational modification of proteins (1, 2). The resulting interdisciplinary field of immunometabolism explores metabolic pathways within immune cells, how these pathways are modulated under immunological stress, and functional consequences at cellular, systemic, and tissue levels (3). Immunological stressors include the aetiology and symptomology of chronic inflammatory diseases such as cardiovascular disease (4), multiple sclerosis (5), and rheumatoid arthritis (6); obesity-, diabetes-, and cancer-induced metabolic dysregulation (7); traumatic injury (8, 9); and physiological stressors such as diet (10) and exercise (11, 12). Immunometabolic research is critical within the wide-ranging contexts of health and disease, holding important applications to the improvement of medical provisions in transplantation (13, 14), oncology (15, 16), pharmacology (17, 18), and metabolic disorders (17, 19).

Peripheral venous blood is one of the most accessible and informative biological fluids used for examination of immunometabolic processes in humans. Peripheral blood mononuclear cells (PBMCs) represent a diverse group of predominantly lymphocytes and monocytes that circulate between lymphatic organs and other tissues to facilitate whole body immune responses. Therefore, PBMCs have been proposed as easily accessible biomarkers with the potential to reflect whole-body bioenergetic health due to their chronic exposure to nutritional and metabolic stimuli in circulation (20–22), hence holding unique value in immunometabolic research. Moreover, upon activation, PBMCs bioenergetic profile is modulated to meet the energetic demands of producing pro-inflammatory cytokines, increasing activation markers, and inducing the cell proliferation and differentiation essential for immune responses to infection or autoimmune conditions. Measuring metabolic fluxes in real-time can therefore provide valuable insights into energy supplying pathways driving immune function and dysregulation in disease. Notably, PBMC bioenergetic dysfunction is associated with chronic metabolic diseases including type 2 diabetes (23), Alzheimer’s (24), hepatic steatosis (25), and chronic inflammation-induced decline in physical function with age and obesity (26); the significance for precise biomarker identification and clinical translation within PBMC bioenergetic research is thus evident. This diagnostic potential has been explored through the bioenergetic health index (BHI), a single-value biomarker of mitochondrial function, calculated from parameters of mitochondrial energy metabolism probed by extracellular flux (XF) analysis (27).

Over the past decade, XF analysis has emerged as a powerful tool for examining perturbations in PBMC bioenergetics from real-time measurements of oxygen consumption rate (OCR) and extracellular acidification rate (ECAR). Combined with modulators of cellular respiration and *ex vivo* activators, these measurements offer critical insights into mitochondrial and glycolytic energy metabolism in different contexts (28, 29). Although PBMCs are readily used for XF analysis (30–32), variability in processing, handling and bioenergetic profiling complicates cross-study comparisons and limits reproducibility. Despite efforts to improve the reliability of XF data using PBMCs (33), standardised protocols for reliably evaluating both mitochondrial and glycolytic parameters within the same cells require further optimisation. For example, the widely recognised ‘Mito Stress Test’ is accepted as the gold standard for assessing mitochondrial function with intact cells, however due to cytotoxic and pH side effects of Carbonyl cyanide 4-(trifluoromethoxy)phenylhydrazone (FCCP), downstream measurements of glycolytic compensation using ECAR or proton efflux rate (PER) are compromised with FCCP. Moreover, glycolytic capacity, which is controlled by cellular energy demands, often exceeds the maximal glycolytic rate achieved with mitochondrial inhibition by rotenone plus antimycin a, as is used in the aforementioned ‘Mito Stress Test’. It is also worth noting that ECAR is a poor indicator of glycolytic metabolism linked to glycolytic lactate efflux due to contributions from mitochondrial CO_2_ acidification and lack of correction for media buffering. To ensure reliability of XF data for profiling both mitochondrial and glycolytic energy metabolism on the same cells, a set of considerations need to be addressed including mitochondrial uncoupler being used, inducers of cellular energy demand, buffer capacity of XF media, and mitochondrial CO_2_ contributions to ECAR. Moreover, external factors like cell handling, processing and plating need to be controlled carefully (33).

The aim of this study was to develop and optimise a single-run XF assay for real-time metabolic profiling of PBMCs and examine the effect of isolation method and blood processing time on metabolic outcomes. By using XF analysis, we optimised PBMC seeding density and developed a combined injection strategy to profile both mitochondrial and glycolytic function of the same cells in the same run. Using this protocol, we examined the effect of isolation method and blood processing time on PBMC mitochondrial respiration and glycolytic activity. Our findings aim to improve the accuracy, reproducibility, and reliability of real-time bioenergetic assessments of PBMCs, particularly when profiling mitochondrial and glycolytic flux simultaneously.

## 2 Material and methods

### 2.1 Ethics

This study was given favourable ethical opinion from the University of Birmingham Science, Technology, Engineering and Mathematics (STEM) Ethical Review Committee (ERN_19-1574AP3). Privacy rights of human donors were observed, and collection and processing of venous blood samples was performed following written informed consent.

### 2.2 Blood sample collection

Blood samples were collected between 09:00am and 10:00am on 4 consecutive days from a healthy 35-year-old male (body mass index = 22.2 kg/m^2^) and after an overnight fast. Each day, 10 mL blood was collected into 2 K_2_EDTA vacutainer tubes (Becton, Dickson & Company, Oxford, UK) by venipuncture – K_2_EDTA vacutainer tubes were used to minimise inter-sample variation (34). Samples were gently inverted several times to prevent coagulation and either processed for PBMC isolation immediately (as for cell seeding density optimisation, section 2.4) or collected over consecutive days to assess the effect of processing time on PBMC bioenergetic parameters. To address the effect of processing time, blood samples were collected consecutively 3, 2, and 1 day(s) prior to processing (herein referred to as 72hr sample, 48hr sample, and 24hr sample respectively) and placed on their side in a marked container for storage in a temperature-controlled laboratory set at 19°C until the day of processing. Blood collected on the morning of the experimental procedure (herein referred to as ‘0hr sample’) was processed immediately, alongside the other samples. Whole blood counts were performed the day of each blood collection using an automated haematology analyser (Yumizen H500, HORIBA Medical, Kyoto, Japan) to examine any potentially significant differences in whole blood parameters between collection days (**Supplementary Table S1**). To mitigate biological interference, PBMCs were isolated from a single donor’s blood throughout this study but assessed multiple times across different months. To examine the individual donor variation in our study we have included coefficient of variation data from all technical replicates across 3 independent blood donations for each metabolic parameter assessed (**Supplementary Figure S1**). To control for illness symptoms that might influence immunity between multiple study visits the ‘Wisconsin Upper Respiratory Symptom Survey’ was also completed each morning prior to blood sampling (35).

### 2.3 Isolation of peripheral blood mononuclear cells from whole blood

PBMCs were isolated from 4mL whole blood using EasySep™ Direct PBMC isolation kits (STEMCELL Technologies, Vancouver, Canada) with “The Big Easy” magnet, or by SepMate™-15 tubes (STEMCELL Technologies) as per the manufacturer instructions. To remove any potential thrombocyte contamination in PBMC isolations using SepMate™ tubes, we added an extra centrifugation step following the initial centrifugation of 120 *x* g for 10 minutes at room temperature, with the brake off. Following isolation, the enriched PBMC suspension was transferred into a 15 mL conical tube and centrifuged at 500 *x g* for 10 minutes. PBMCs were washed in 5 mL pre-warmed XF media (Seahorse XF RPMI pH 7.4, supplemented with 10mM glucose, 2mM L-glutamine and 1mM sodium pyruvate), before finally resuspending into 1 mL XF media for counting. Viable PBMCs were counted automatically after staining with acrid orange and propidium iodide using a Cellometer Auto 2000 Cell Counter (Nexcelom Bioscience, Massachusetts, USA) and used for downstream assays (**Supplementary Figure S2**).

### 2.4 Optimising PBMC seeding density for accurate assessment of real-time metabolic profiling using XF analysis

Real-time metabolic profiling of intact cells refers to measurements of both oxidative and glycolytic flux to establish mitochondrial and glycolytic energy metabolism from the same cells, respectively. Measurements of cellular bioenergetics using XF analysis are becoming more mainstream, however the cell seeding density and thus cell confluency influences calculations of both OCR and ECAR for assessing metabolic profiles of cells. We assessed this by seeding freshly isolated PBMCs into XFe96 V3 PS microwell plates (Agilent Technologies) pre-coated with Cultrex poly-D-lysine (#3439-100-01, bio-techne, R&D systems) at densities of 0.5x10^5^, 1x10^5^, 2x10^5^ or 4x10^5^ cells per well. To improve plating efficiency between wells, cells were counted after centrifugation and diluted to the necessary cell seeding density and volume prior to seeding with an automated multi-stepper pipette (Voyager, Integra Biosciences, UK). Cells were initially seeded in a volume of 80µL per well and then centrifuged at 100 x g for 1 minute without breaking to facilitate cellular adhesion. After incubation at 37°C under air for 15 minutes, the experimental well volume was increased to 180µL by addition of XF media. 180µL/well of XF media was added to background control wells (A1, H1, A12, H12). Experimental wells were visually inspected with an inverted light microscope for even cell seeding. Basal OCR and ECAR of PBMCs were assessed using an XFe96 analyser (Agilent Technologies, USA). Each measurement cycle consisted of a 3-minute mix and 3-minute measure period. For measurements of glycolytic metabolic profiling, ECAR were converted to proton efflux rates (PER) using Seahorse Analytics (Agilent Technologies) by considering the buffering capacity of XF RPMI media.

### 2.5 Real-time metabolic profiling of PBMC using XF analysis

PBMCs were seeded into XFe96 V3 PS microwell plates as described in section 2.4 at 2x10^5^ cells per well. To stimulate antigen presenting T cells within the PBMC fraction, wells were treated with 20µL/well ImmunoCult™ Human CD3/CD28/CD2 T Cell Activator (STEMCELL technologies, #10970) and left at 37°C under air for 30 minutes prior to being assayed in the XF analyser. Following 4 baseline measurement cycles, the sequential injection of oligomycin (2 µg/mL, Sigma-Aldrich, catalogue #O4876), BAM15 (3 µM, bio-techne, catalogue #5737), Rotenone (2 µM, Sigma-Aldrich, catalogue #R8875) plus Antimycin A (2 µM, Sigma-Aldrich, catalogue #A8674), and monensin (25 µM, Sigma-Aldrich, catalogue #M5273) were added to establish ATP-coupled respiration, maximal respiratory capacity, non-mitochondrial respiration, and maximal glycolysis, respectively (**Supplementary Figure S3**). Each measurement cycle consisted of a 3-minute mix and 3-minute measure period.

### 2.6 Data analysis

Mitochondrial and glycolytic bioenergetic parameters were calculated from OCR and PER as previously determined (36). Glycolytic PER (glycoPER), an accurate measure of glycolysis, was obtained by subtracting mitochondrial PER from total PER using a pre-determined H^+^/O_2_ value of 0.38 as empirically calculated recently by Desousa et al. (37). ATP synthesis rates were calculated as described previously (37–39). Mitochondrial control analysis including respiratory control ratio (RCR), BHI and mitochondrial toxicity index (MTI) were calculated from mitochondrial bioenergetic parameters. All advanced data analysis calculations and defined formulas are detailed in **Supplementary Table S2**. Statistical analyses were performed using GraphPad Prism Version 9.5.1 for Mac OS X (San Diego, CA, USA). Linear regression analysis was performed for PBMC seeding density optimisation. Two-way ANOVA was performed for all bioenergetic parameters with multiple comparison *post hoc* tests (detailed in figure legends) performed when a significant interaction effect was present. Data are presented as *mean ± SEM*, unless otherwise indicated. P-values < 0.05 were considered statistically significant.

## 3 Results

### 3.1 Identifying optimal seeding densities of PBMCs for reliable measurements of cellular bioenergetics using XF analysis

**Figure 1:**
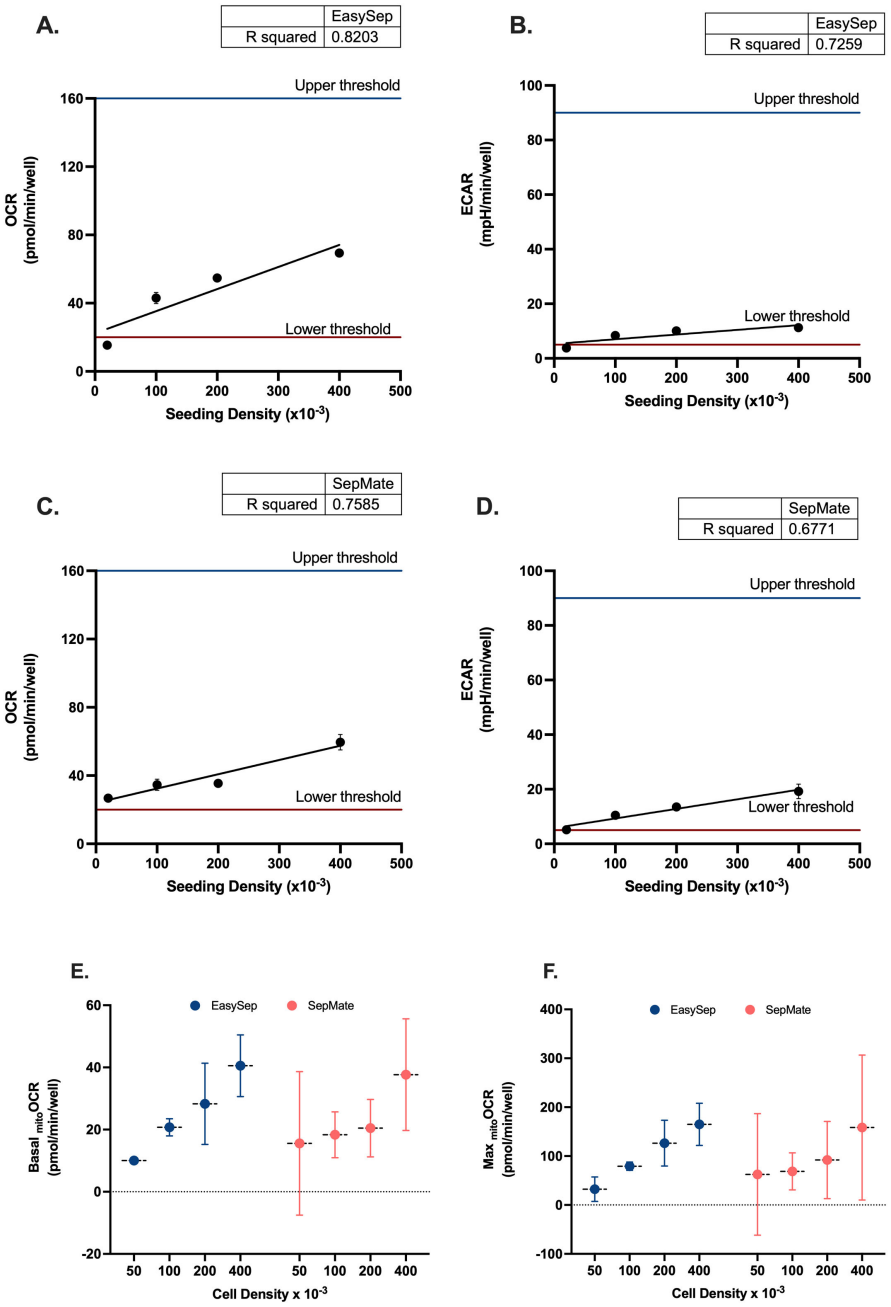
Seeding density optimisation for PBMC bioenergetic analysis using Seahorse XF technology. Linear regression analysis of oxygen consumption rate (OCR) versus seeding density for **(A)** EasySep™-isolated and **(C)** SepMate™-isolated PBMCs. Corresponding extracellular acidification rate (ECAR) analysis for **(B)** EasySep™ and **(D)** SepMate™ isolation methods. Upper and lower threshold lines indicate acceptable measurement ranges. Comparative analysis of **(E)** basal mitochondrial OCR and **(F)** maximal mitochondrial OCR across four seeding densities (50k, 100k, 200k, 400k cells/well) for both isolation methods. Data in panels A-D represent single experimental runs with 4–5 technical replicates per density. Data in panels E-F represent mean ± CV from three independent experiments with 6 experimental replicates each. Blue circles indicate EasySep™-isolated cells; red circles indicate SepMate™-isolated cells. R² values indicate goodness of fit for linear regression models. OCR and ECAR values are expressed as pmol/min/well and mpH/min/well, respectively.

Due to the lower and upper sensitivity ranges of XF analysis it is important to determine optimal cell seeding densities that align with target baseline ranges for the XF analyser being used to achieve reliable measurements of OCR and ECAR. For XFe96 analysers, the manufacturer recommended detection limit of OCR is between 20 and 160 pmol/min/well, whilst the detection limit of ECAR is between 5 and 90 mph/min/well. To determine the optimal number of PBMCs per well for XF analysis using the XFe96 analyser, PBMCs isolated by either density gradient (SepMate™) or magnetic separation (EasySep™ Direct) were seeded at densities of 0.5x10^5^, 1x10^5^, 2x10^5^, and 4x10^5^ and assessed for basal OCR and ECAR. Basal OCR and ECAR of PBMCs isolated by either SepMate™ or EasySep™ Direct seeded at densities of 0.5x10^5^ were close to or below the OCR and ECAR lower thresholds of 20 pmol/min/well and 5 mph/min/well, respectively (**Figures 1A-D**). Although upper limits of baseline target ranges for OCR and ECAR at the higher seeding density of 4x10^5^ cells were not met, a mild attenuation of the linear proportional relationship between seeding density and OCR or ECAR was apparent (**Figure 1**).

Comparative analysis of basal and maximal mitochondrial OCR across the four seeding densities revealed superior performance consistency with EasySep™ isolation (**Figures 1E, F**). EasySep™-isolated PBMCs demonstrated progressively increasing basal mitochondrial OCR from 10.0 ± 2.4 pmol/min/well at 50k density to 40.5 ± 4.0 pmol/min/well at 400k density, with relatively tight error margins indicating good reproducibility. In contrast, SepMate™-isolated cells showed greater variability, particularly at lower seeding densities, with basal OCR ranging from 15.6 ± 9.3 pmol/min/well (50k) to 37.7 ± 7.2 pmol/min/well (400k). Maximal OCR patterns followed similar trends, with EasySep™ consistently achieving higher absolute values and better reproducibility (32.4 ± 10.1 to 164.9 ± 17.3 pmol/min/well) compared to SepMate™ (62.5 ± 50.0 to 158.3 ± 59.7 pmol/min/well). 100k and 200k seeding densities appear as optimal for both methods, providing the best balance between signal strength and measurement precision, with EasySep™ showing particularly robust performance at these densities. These observations are further supported by coefficient of variation analysis (**Supplementary Table S3**.0), which confirmed that EasySep™ consistently achieved excellent (CV <10%) or good (CV 10%-20%) reproducibility for both basal and maximal parameters at 100k and 200k densities, while SepMate™ showed higher variability (CV% >20%) and fair to poor reproducibility for one or both mitochondrial parameters at all seeding densities.

### 3.2 Developing an injection strategy to accurately measure mitochondrial and glycolytic energy metabolism of PBMCs

**Figure 2:**
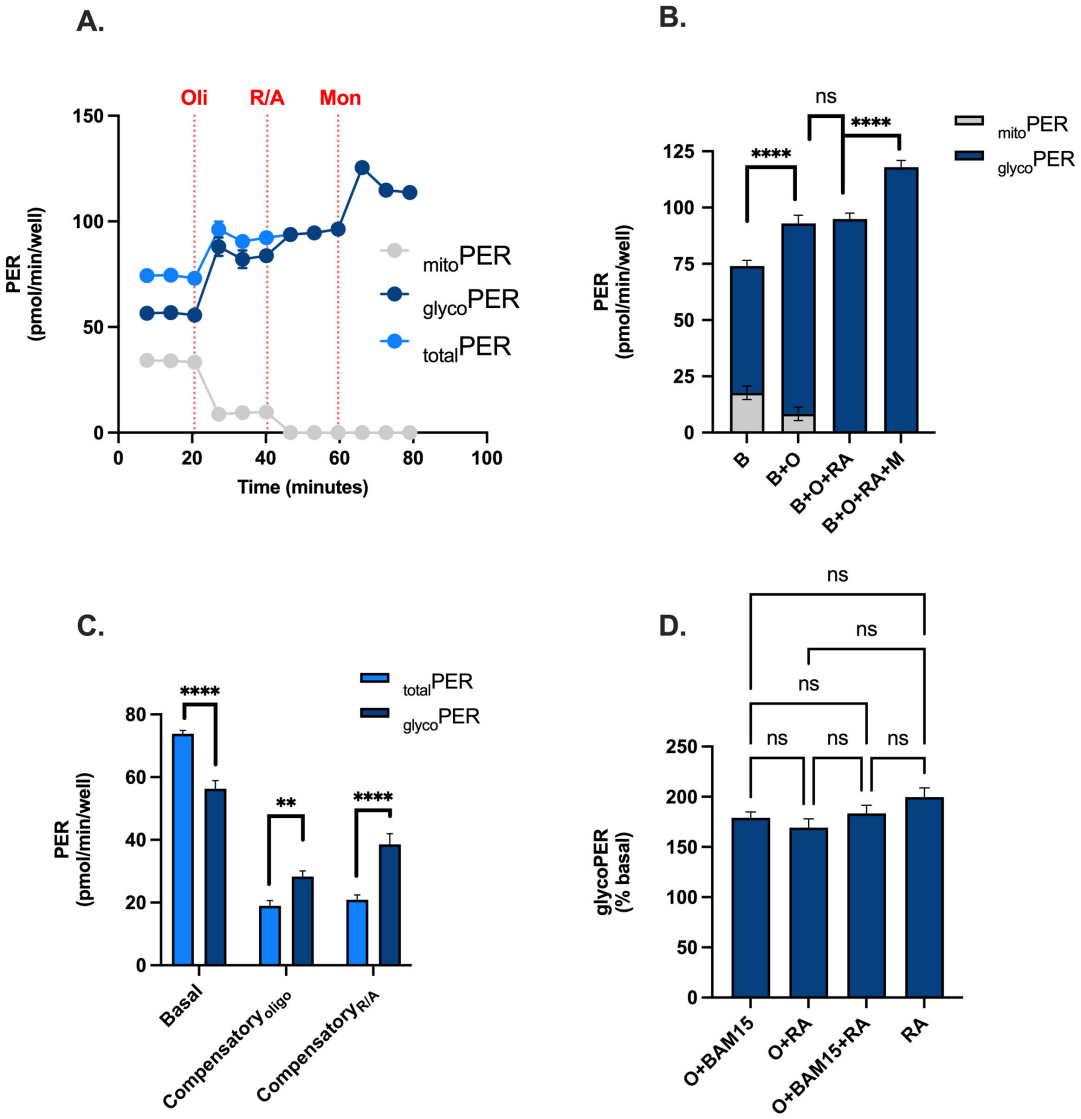
Feasibility of using a modified injection strategy for real-time metabolic profiling of PBMCs. PBMCs were assessed for OCR, PER, mitoPER and glycoPER in the presence and absence of the uncoupler BAM15, mitochondrial inhibitors (oligomycin or rotenone + antimycin A), and the ionophore monensin. **(A)** Kinetic traces of total PER (light blue circles), glycoPER (dark blue circles) or mitoPER (grey circles) following the sequential addition of respiratory effectors oligomycin, ronteone plus antimycin a and monensin. **(B)** Contributions of glycoPER (dark blue bars) and mitoPER (grey bars) from PBMCs in the presence of respiratory effectors. **(C)** Basal and compensatory PER (light blue bars) and glycoPER. **(D)** Respiratory effector-induced glycoPER (% basal). Data represent mean ± SEM from 3 technical replicates. Statistical differences were tested for by One-Way ANOVA with Tukey’s *post-hoc* test (**P < 0.01, ****P<0.0001), ns = not significant.

To identify if XF analysis could be used to metabolically profile PBMCs in real-time using a standardised assay workflow, we analysed rates of OCR and PER in the presence and absence of the uncoupler BAM15. Given the contribution of mitochondrial oxidative phosphorylation for ATP supply in PBMCs, we showed that mitochondrial respiratory activity contributes significantly to proton efflux rate (_mito_PER) when measured with XF analysis (**Figures 2A, B**). As a result, _mito_PER accounted for 24% and 9% of _total_PER in the absence and presence of oligomycin respectively. Without correcting appropriately for _mito_PER, PER associated with glycolytic lactate formation (_glyco_PER) is significantly overestimated by 31% under basal conditions (**Figure 2C**). Moreover, compensatory effects of glycolysis in response to mitochondrial respiratory inhibition with oligomycin or Rotenone plus Antimycin A is significantly underestimated by 32% and 81% respectively (**Figures 2A, C**). We also examined the effect of the ionophore monensin to identify if increased ATP demand (through activation of Na/K-ATPases) increased glycolytic flux beyond compensatory adjustments following mitochondrial respiratory inhibition with Rotenone plus Antimycin A as reported previously (38). After correcting appropriately for _mito_PER, monensin increased _glyco_PER from 95 pmol/min/well in the presence of oligomycin and 118 pmol/min/well in the presence of rotenone plus antimycin A (**Figure 2B**), consistent with the literature (32). As expected, the addition of oligomycin in the presence or absence of BAM15 in our injection strategy had no significant effect on Rotenone plus Antimycin A-induced _glyco_PER (**Figure 2D**). These data confirm that our modified injection strategy using oligomycin, BAM15, Rotenone plus Antimycin A and monensin provides OCR and PER data that can be used for downstream bioenergetic analysis to metabolically profile PBMCs from the same wells of an XFe96 microwell plate (**Supplementary Figure S3**).

### 3.3 Isolation protocol and blood processing time reveals differences in cellular bioenergetics of PBMCs

**Figure 3:**
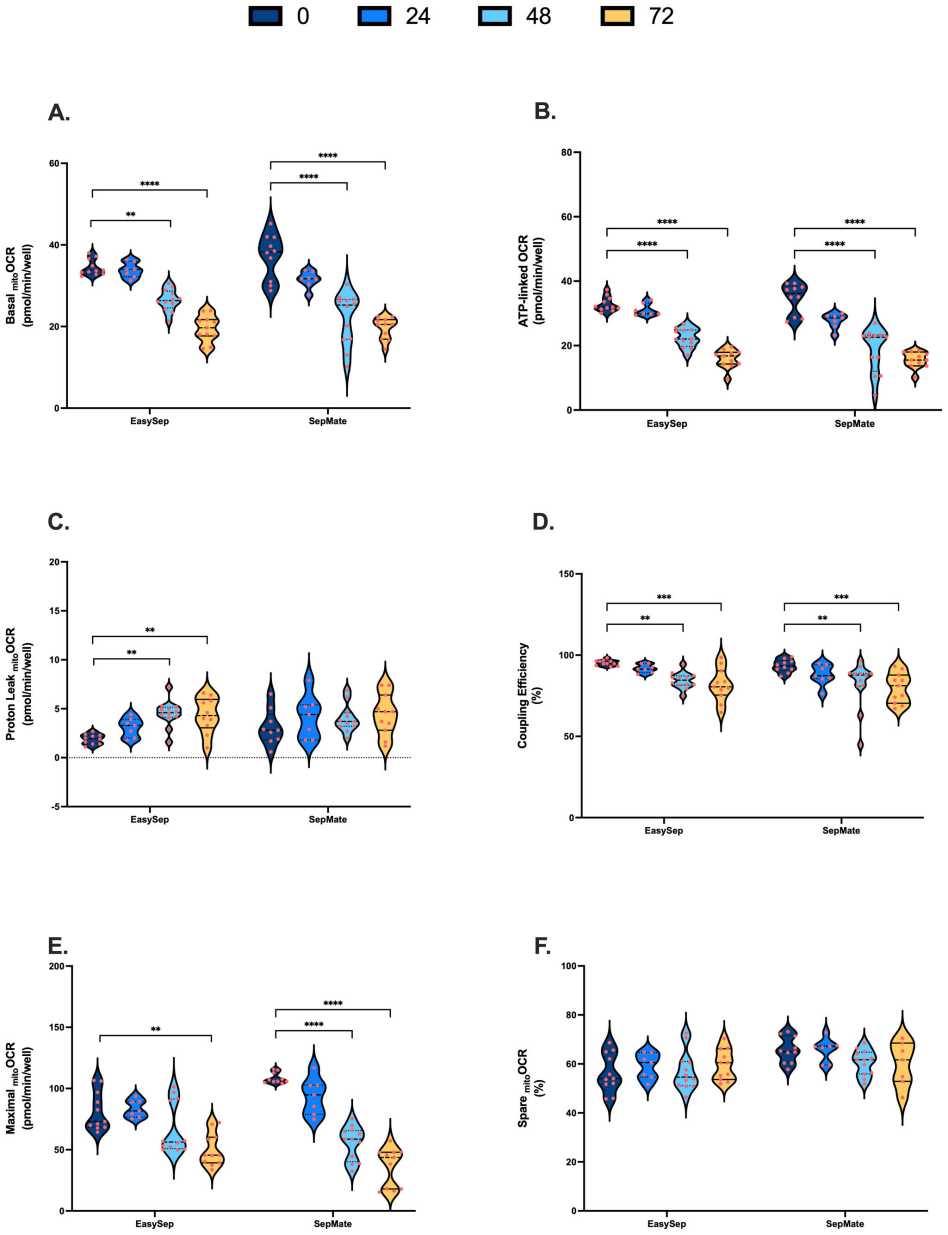
Impact of blood processing time and isolation method on PBMC mitochondrial energy metabolism. PBMCs isolated from 0-hour, 24-hour, 48-hour, or 72-hour rested blood using SepMate™ or EasySep™ Direct protocols were metabolically profiled in real-time using XF analysis to assess mitochondrial function. **(A)** Basal mitochondrial respiration (pmol O_2_/min/well). **(B)** Mitochondrial respiration (pmol O_2_/min/well) coupled to ATP synthesis. **(C)** Mitochondrial respiration (pmol O_2_/min/well) dissipated as heat due to proton leak. **(D)** Coupling efficiency of oxidative phosphorylation expressed as percent of baseline mitochondrial respiration. **(E)** Maximal mitochondrial respiratory (pmol O_2_/min/well) capacity. **(F)** Spare respiratory capacity presented as percentage of maximal mitochondrial respiratory capacity. Data represent means ± SEM from 8–12 technical replicates across three independent experiments. Statistical differences were tested for by Two-Way ANOVA with Dunnett’s *post-hoc* test (**P < 0.01, ***P < 0.001, ****P<0.0001).

Using our metabolic profiling test described in 3.2, mitochondrial and glycolytic energy metabolism of PBMCs isolated from 0hr, 24hr, 48hr or 72hr rested blood samples using SepMate™ or EasySep™ Direct isolation protocols (**Figures 3A, B**) were probed with XF analysis. All metabolic parameters are presented as violin plots to provide complete visualization of data distribution and individual measurement variability.

PBMCs isolated from blood rested for 48hrs and 72hrs exhibited significantly decreased rates of basal, ATP-linked and maximal mitochondrial respiration (**Figures 3A, B, E**). Proton leak in PBMCs isolated from 48hr and 72hr rested blood was increased, albeit not significantly from SepMate™ isolations (**Figure 3C**). Consistent with decreased ATP-linked respiration (**Figure 3B**) and increased proton leak (**Figure 3C**), coupling efficiency of oxidative phosphorylation significantly decreased by ~15% in PBMCs isolated from blood left for 48 and 72 hrs when compared to freshly isolated PBMCs (**Figure 3D**). Despite significant decreases in maximal mitochondrial respiration (**Figure 3E**), when expressed as % max, spare respiratory capacities of PBMCs were not altered by blood processing time (**Figure 3F**). Although decreases in basal and maximal mitochondrial oxygen uptake were greater in PBMCs isolated using SepMate™ from the 48 and 72 hr blood samples, no significant differences in mitochondrial respiratory parameters were observed between isolation method (**Figure 3**).

**Figure 4:**
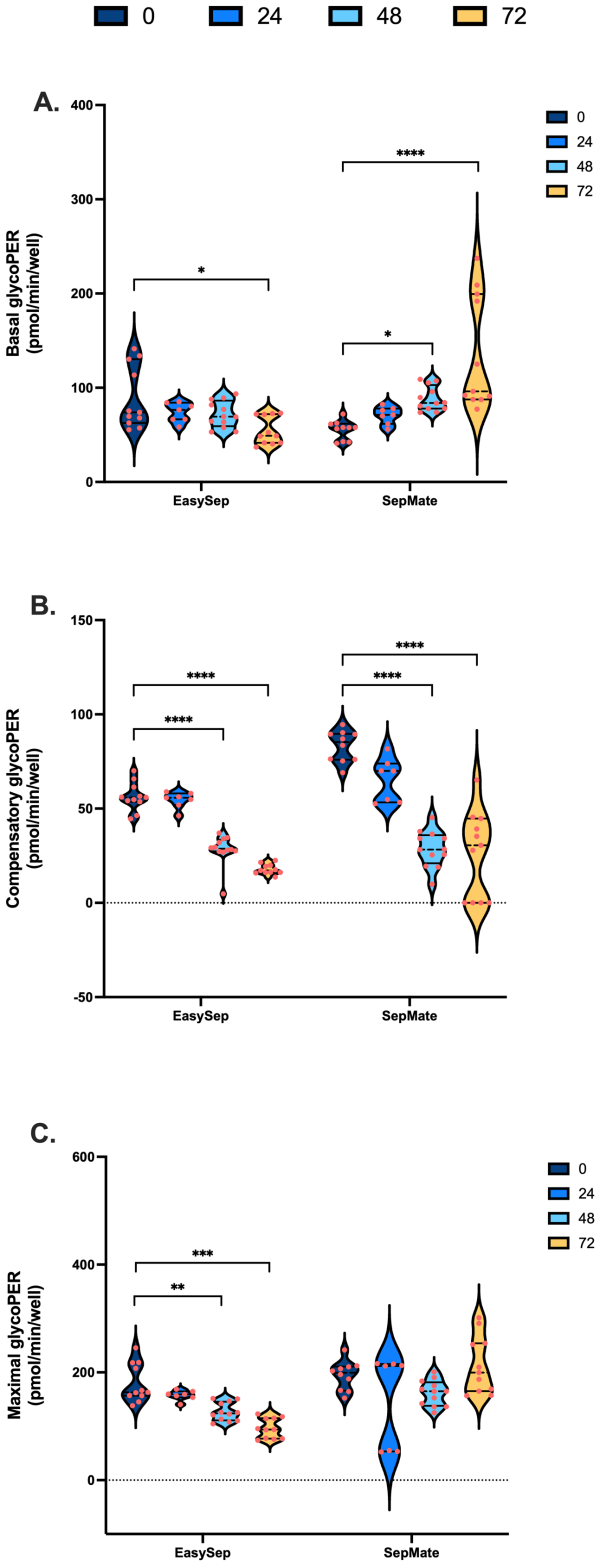
Impact of blood processing time and isolation method on PBMC glycolytic energy metabolism. PBMCs isolated from 0-hour, 24-hour, 48-hour, or 72-hour rested blood using SepMate™ or EasySep™ protocols were metabolically profiled to assess glycolytic function. **(A)** Basal glycolytic PER (pmol/min/well). **(B)** Oligomycin-induced compensatory glycolytic PER (pmol/min/well). **(C)** Maximal glycolytic PER (pmol/min/well) with monensin. Data represent means ± SEM from 8–12 technical replicates across three independent experiments. Statistical differences were tested for by Two-Way ANOVA with Tukey’s *post-hoc* test (**P < 0.01, ***P < 0.001, ****P<0.0001).

To examine the effect of blood processing time and isolation method on PBMC glycolysis, glycoPER was calculated after correction of total PER for mitochondrial PER contributions as described in **Supplementary Table S2**. Blood processing time significantly increased basal glycoPER from PBMCs isolated by SepMate™ but not EasySep™ Direct isolation method (**Figure 4A**). Interestingly, basal glycolysis was significantly decreased from 105 pmols/min/well to 63 pmols/min/well in PBMCs isolated from blood rested for 72 hrs using EasySep™ Direct (**Figure 4A**). Consistent with decreases in ATP-coupled respiration (**Figure 3B**), compensatory glycolysis was significantly lower in PBMCs isolated from blood left for 48 or 72 hrs vs. 0 hrs (**Figure 4B**). Interestingly, glycolytic capacity decreased significantly in PBMCs isolated from blood rested for 48 or 72 hrs using EasySep™ Direct but not SepMate™ isolation method (**Figure 4C**). Unlike the case with mitochondrial energy metabolism, both basal (Interaction P = 0.0001) and maximal (Interaction P = 0.0017) glycolysis in PBMCs between EasySep™ Direct or SepMate™ methods were significantly different (**Figures 4A, C**). The superior consistency of EasySep™ Direct isolation was evident in inter-donor variability analysis, which revealed CV% values typically below 25% for most parameters across all time points. In contrast, SepMate™ isolation showed increased variability, particularly for glycolytic measurements at extended processing times (CV% >30% for some parameters at 72 hours) (**Supplementary Figure S1**). Of importance, differences in glycolytic flux between isolation method were likely due to red blood cell contamination in PBMC isolations using the SepMate™ isolation method, which increased with increased time to processing of sample. This was corroborated by visualisation of red blood cell contamination in XF microplate wells seeded with PBMCs isolated from blood left for 48 and 72 hrs (**Supplementary Figure S3**). This red blood cell contamination likely contributed to the increased measurement variability observed with SepMate™ isolation, reinforcing the methodological advantages of EasySep™ Direct for extended processing times. To try and correct for the red cell contamination in PBMC isolations using SepMate™ from blood left for 48 and 72 hours we used ammonium chloride (AC) as a red cell lysis agent to lyse red blood cells from these PBMC isolations (**Supplementary Figure S4**). However, AC had adverse effects on the respiratory control ratio of PBMCs when compared to PBMCs isolated without the use of AC (**Supplementary Figure S4B**). This was attributed to further decline of oxidative respiratory capacity (**Supplementary Figure S4C**). We also show that AC lowers stimulation-induced lactate efflux of T-lymphocytes in PBMC isolations using the CD28/CD3 activator (**Supplementary Figure S4D**).

**Figure 5:**
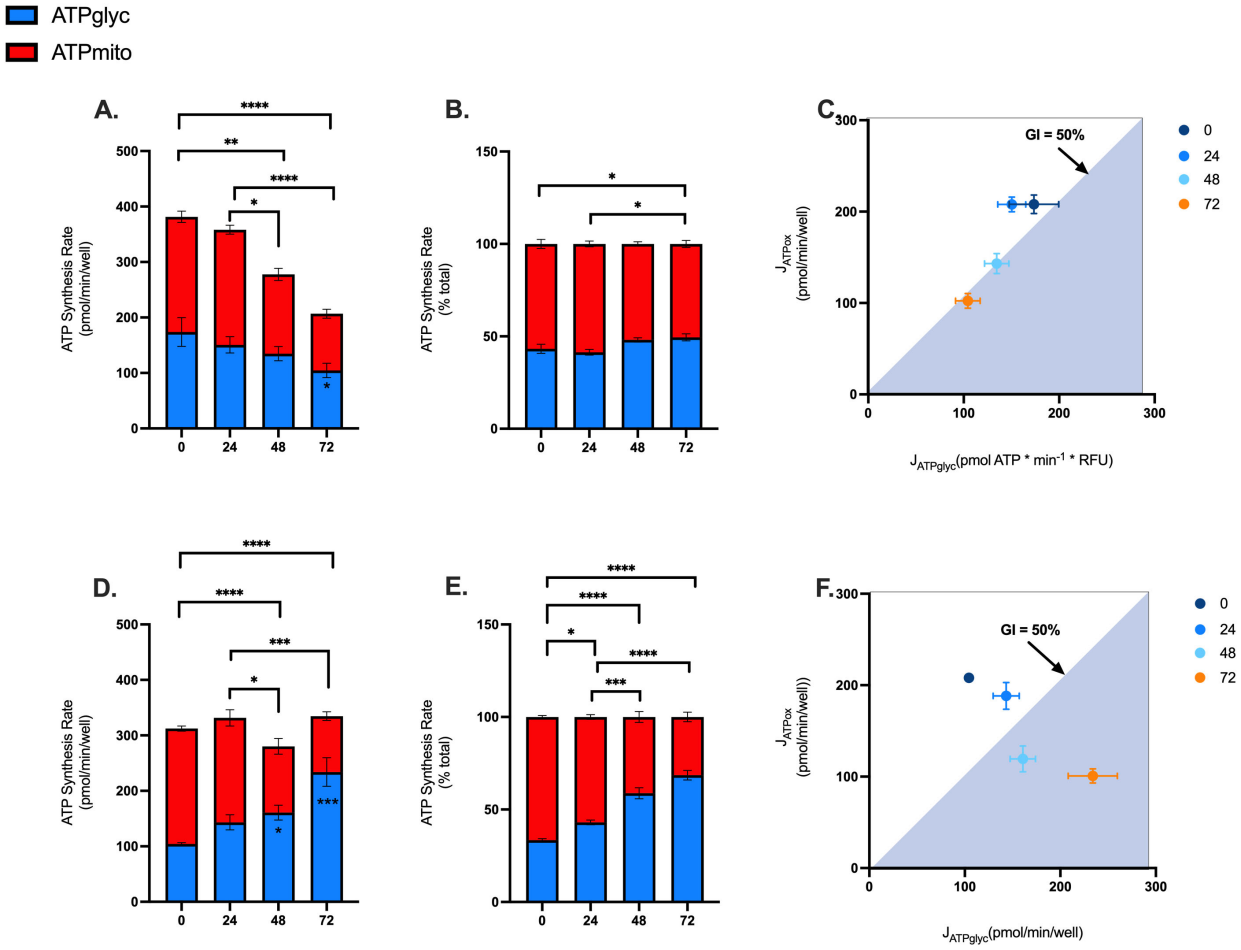
Effects of blood processing time and isolation method on PBMC ATP supply flux. Mitochondrial and glycolytic ATP supply rates were estimated from OCR and PER data using XF analysis. Mitochondrial (red fraction) and glycolytic (blue fraction) ATP synthesis rates from PBMCs isolated by EasySep Direct™ **(A)** or SepMate™ **(D)** at different blood processing times. Mitochondrial (red fraction) and glycolytic (blue fraction) ATP synthesis rates expressed as percentage of total ATP supply flux from PBMCs isolated by EasySep Direct™ **(B)** or SepMate™ **(E)** at different blood processing times. XY scatter graph of ATP_mito_ (pmol/min/well) expressed as a function of ATP_glyc_ (pmol/min/well) from PBMCs isolated by EasySep Direct™ **(C)** or SepMate™ **(F)**. A glycolytic index (GI) calculated as 100 x ATP_glyc_/total ATP) of 50% or more denotes the threshold for a primarily glycolytic cell, points with a 50% GI or more lye within the blue shaded area **(C, F)**. Data represent means ± SEM from 8–12 technical replicates across three independent experiments. Statistical differences were tested for by Two-Way ANOVA with Sidak’s *post-hoc* test (*P < 0.05, **P < 0.01, ***P < 0.001, ****P<0.0001).

Given the effects of blood processing time on mitochondrial and glycolytic function of PBMCs (**Figures 3** and **4**), we calculated rates of ATP synthesis to better understand the extent to which blood processing time and/or isolation method influences PBMC bioenergetics. Consistent with effects on mitochondrial respiration, rates of mitochondrial ATP supply were significantly lower in PBMCs isolated from blood rested for 48 and 72 hrs (**Figures 5A, D**). Moreover, glycolytic ATP was significantly decreased in PBMCs isolated from blood rested for 72 hrs using EasySep™ Direct (**Figure 5A**), but significantly increased in PBMCs isolated from blood rested for 48 and 72 hrs using SepMate™ (**Figure 5D**). In addition to decreased rates of ATP synthesis, when glycolytic and mitochondrial ATP are expressed as % total ATP, it is revealed that increased blood processing time increases the glycolytic index of PBMCs isolated by EasySep™ Direct (**Figure 5B**) and SepMate™ (**Figure 5E**) methods. This is even more evident when mitochondrial ATP (JATPox) is expressed as a function of glycolytic ATP (JATPglyc), whereby PBMCs isolated from blood rested for 48 and 72 hrs have higher glycolytic indexes compared with those isolated from 0hr or 24hr blood (**Figures 5C, F**). This is even more evident in PBMCs isolated by SepMate™ (**Figure 5F**), consistent with the notion that increased red blood cell contamination contributes to glycolytic metabolism (**Supplementary Figure S2**).

### 3.4 Advanced analysis of real-time metabolic profiles from PBMCs reveals significant effects of blood processing time on mitochondrial respiratory capacity

To further explore a possible mechanism of action and quantify the magnitude of change in mitochondrial function that occurs in PBMCs isolated from blood rested for 48 and 72hrs, we applied calculations (**Supplementary Table S2**) to determine mitochondrial respiratory control including respiratory control ratio (RCR), BHI, and mitochondrial toxicity index (MTI). The, a composite metric that integrates multiple mitochondrial parameters, has been proposed as a clinically relevant biomarker for assessing mitochondrial dysfunction in blood-based cells (27). The MTI provides quantitative assessment of mitochondrial toxicity by examining the changes in oxidative capacity and coupling control, while RCR evaluates the efficiency of mitochondrial ATP synthesis.

**Figure 6:**
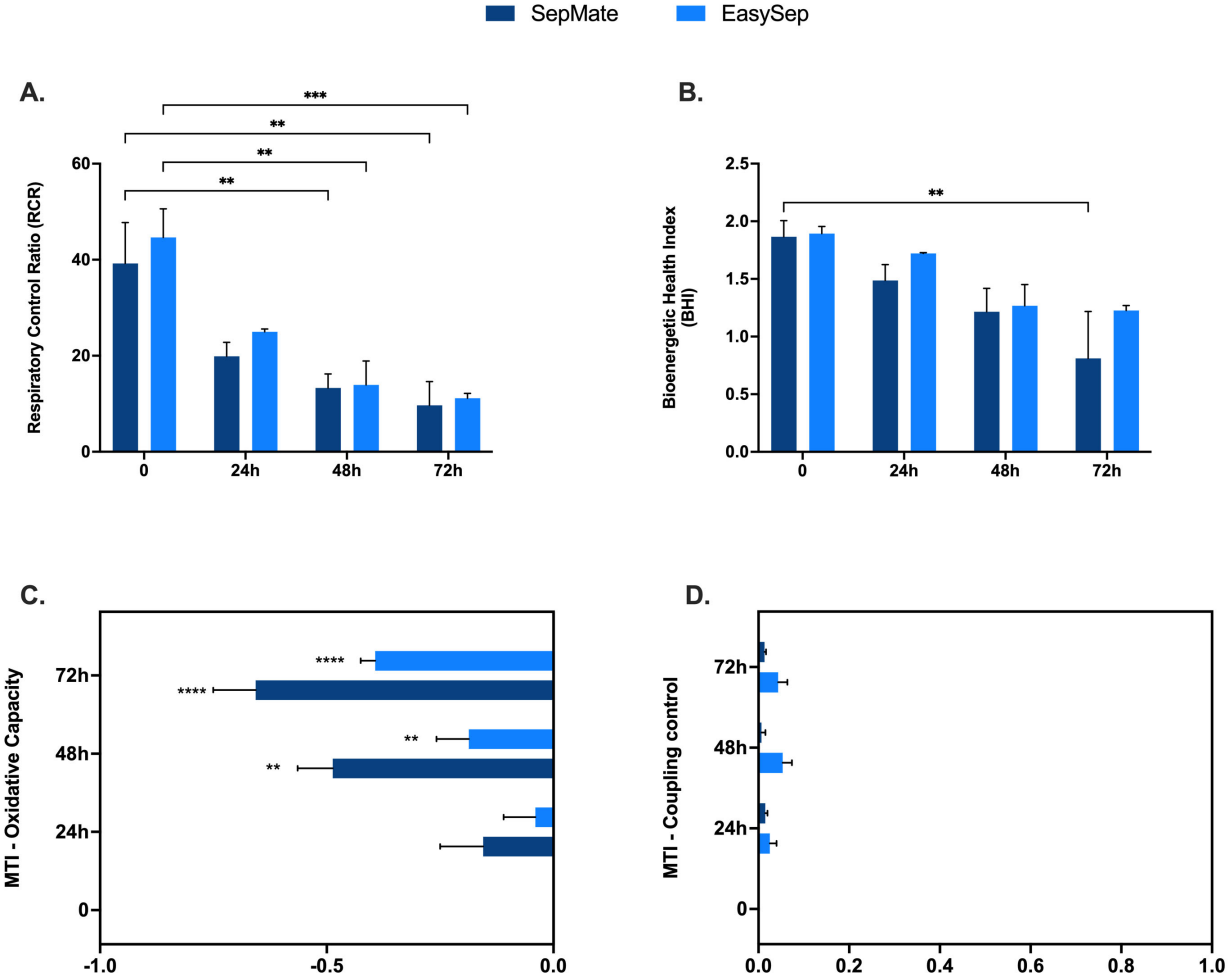
Mechanistic insights into effects of blood processing time on PBMC mitochondrial function. Advanced metabolic control analysis including respiratory control ratio **(A)**, bioenergetic health index (BHI) and mitochondrial toxicity index c of PBMCs isolated from blood rested for 0hr, 24hr, 48hr or 72hr from different isolation method (EasySep™ or SepMate™). Data represent means ± SEM from 8–12 technical replicates across three independent experiments. Statistical differences were tested for by Two-Way ANOVA with Dunnett’s **(A, B)** or Tukey’s **(C, D)** *post-hoc* test (**P < 0.01, ***P < 0.001, ****P<0.0001).

Decreases in the RCR highlights a significant effect of blood processing time on PBMC mitochondrial energy metabolism (**Figure 6A**) and confirms mitochondrial respiratory defects shown in **Figure 3**. Interestingly, the BHI was only significantly different between PBMCs isolated from blood between 0hr and 72hr using SepMate™ (**Figure 6B**). This differential response pattern contrasted with more traditional mitochondrial parameters, such as coupling efficiency and respiratory capacity (**Figures 3D, E**), which demonstrated clear significant differences with extended blood processing time. The selective sensitivity of the BHI compared to individual mitochondrial parameters suggests that while mitochondrial coupling and respiratory capacity are substantially affected by prolonged blood storage, the overall integrated mitochondrial profile remains relatively preserved until more extended storage periods.

Quantification of the MTI revealed that increased blood processing time causes a significant change in mitochondrial oxidative capacity, with little effect on coupling control (**Figures 6C, D**). These findings demonstrate that different analytical approaches to assessing mitochondrial function exhibit varying sensitivities to pre-analytical variables, which has important implications for selecting appropriate metrics in PBMC bioenergetic studies depending on the experimental context and clinical application.

### 3.5 Effect of blood processing time on T cell activation

**Figure 7:**
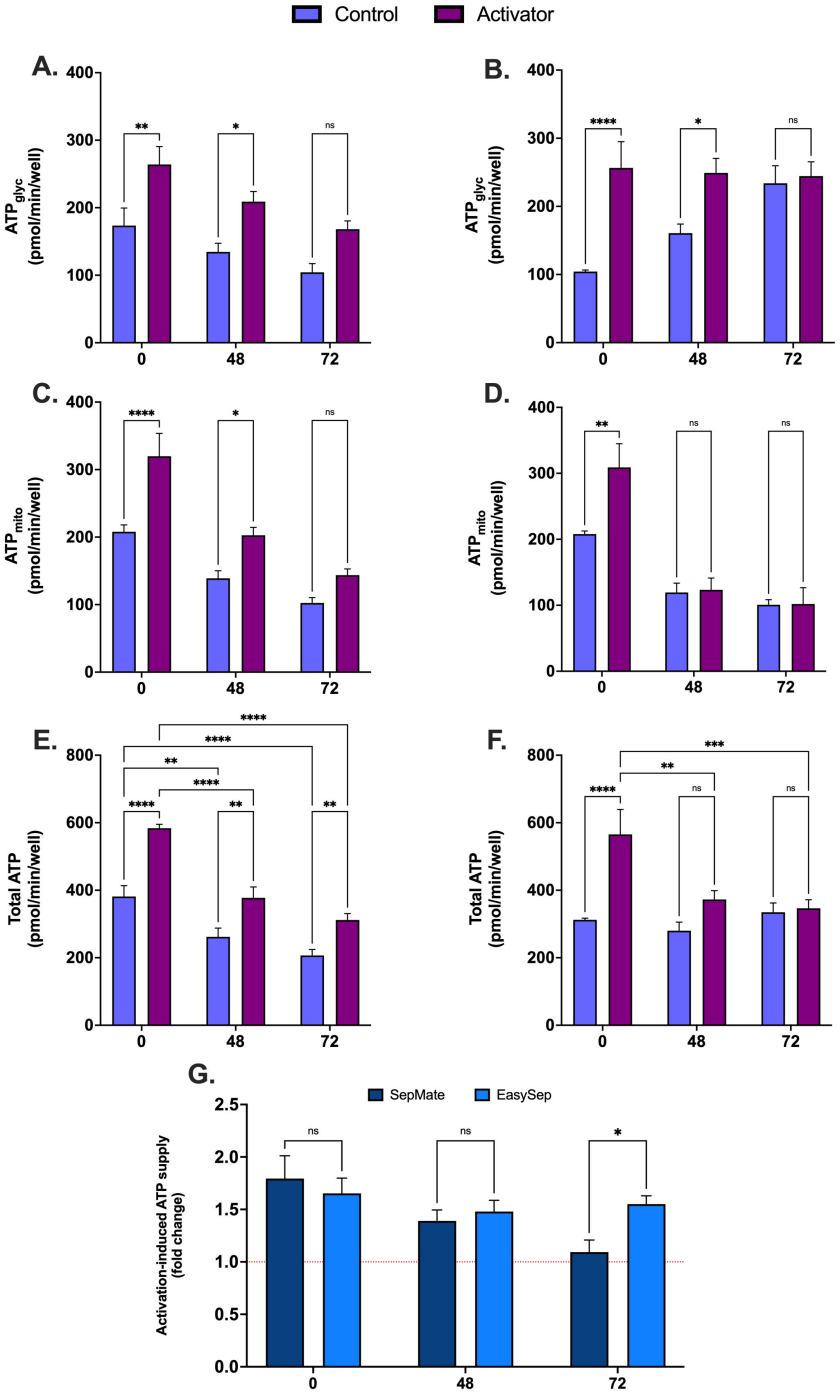
Effect of blood processing time and isolation method on metabolic changes during immune cell activation in PBMCs. Real-time metabolic activity was assessed in PBMCs isolated with EasySep™ direct or SepMate™ methods from whole blood at 0hr, 48hr or 72hr, and treated with (purple bars) or without (blue bars) CD3/CD28 immunocult T-cell activator. Glycolytic ATP supply from PBMCs ± CD3/CD28 immunocult T-cell activator isolated by EasySep Direct™ **(A)** or SepMate™ **(B)**. Mitochondrial ATP supply from PBMCs ± CD3/CD28 immunocult T-cell activator isolated by EasySep Direct™ **(C)** or SepMate™ **(D)**. Total ATP supply from PBMCs ± CD3/CD28 immunocult T-cell activator isolated by EasySep Direct™ **(E)** or SepMate™ **(F)**. **(G)** Activation-induced ATP supply expressed as fold change from total ATP supply rate of PBMCs not treated with CD3/CD28 immunocult T-cell activator. Data represent means ± SEM from 8–12 technical replicates across three independent experiments. Statistical differences were tested for by Two-Way ANOVA with Sidak’s *post-hoc* test (*P < 0.05, **P < 0.01, ***P < 0.001, ****P<0.0001).

To assess whether blood processing time alters metabolic adaptations that drive T cell activation, PBMCs isolated using EasySep™ Direct or SepMate™ from 0hr blood or blood stored for 48 and 72 hours were activated with CD3/CD28 immunocult T-cell activator and profiled using XF analysis. Except for isolated PBMCs from blood stored for 72hrs, activation of antigen presenting T cells in isolated PBMCs resulted in a significant increase in glycolytic ATP supply compared to non-activated PBMCs (**Figures 7A, B**). Typical of T cell activation, mitochondrial ATP supply was significantly increased during activation, but only from PBMCs isolated from blood within 48hrs by EasySep™ Direct (**Figures 7C, D**). While early activation led to significant upregulation of total ATP supply from PBMCs isolated from EasySep™ Direct (**Figure 7E**), total ATP supply was still markedly diminished after 48 and 72 hours of blood storage (P < 0.0001), indicating a critical loss in bioenergetic function with delayed processing (**Figure 7E**). Moreover, PBMCs isolated using SepMate™ exhibited lower activation-induced ATP supply rates compared to EasySep™ Direct isolated PBMCs (**Figure 7G**), which was significant from 72hr rested blood (P < 0.05). This decrease was attributed to a combination of diminished activation-induced mitochondrial oxidative ATP supply (**Figure 7D**) and increased basal glycolytic ATP supply (**Figure 7B**), which may, in part, be due to contamination with red blood cells in the SepMate™-isolated PBMC preparations from blood left for 72 hrs (**Supplementary Figure S3**).

### 3.6 Best practice recommendations for PBMC metabolic profiling

To provide practical guidance for implementing optimised PBMC metabolic profiling protocols, we have summarised our key findings into evidence-based recommendations (**Tables 1**–**3**). Our data demonstrate that EasySep™ Direct isolation consistently achieves better reproducibility with coefficient of variation values below 10% for primary mitochondrial endpoints when blood is processed within 24 hours and cells are seeded at optimal densities (1-2 × 10^5^ cells per well). In contrast, SepMate™ isolation showed acceptable performance for immediate processing but exhibited deterioration in measurement consistency at extended processing times, with CV values >20% for most bioenergetic parameters after 48 hours of blood storage. Our method selection guide reveals a clear decision tree: either isolation method is suitable for immediate processing (<24 hours), EasySep™ Direct is preferred for processing delays of 24–48 hours, and processing beyond 72 hours should be avoided regardless of method. Additionally, our analysis of key biomarkers revealed that while respiratory control ratio serves as a sensitive indicator of processing-induced metabolic changes, the bioenergetic health index showed limited sensitivity to early dysfunction. These standardised parameters, combined with our troubleshooting guide for common technical issues, provide a comprehensive framework for improving the reliability of PBMC metabolic profiling that addresses methodological variability that often limits cross-study comparisons in immunometabolism research.

**Table 1:** Best practice recommendations for PBMC metabolic profiling based on experimental findings - protocol parameters.

Parameter	Optimal recommendation	Supporting evidence from current study
Blood Processing Time	≤24 hours at room temperature (~19 °C).	Maintained bioenergetic parameters and T-cell activation capacity.
PBMC Isolation Method	EasySep™ Direct for processing >24h.	CV <20% for primary endpoints vs. >20% with SepMate™.
Cell Seeding Density	1-2 × 10^5^ cells per well (XFe96).	Optimal detection range with linear OCR/ECAR responses. Excellent or good reproducibility of basal and maximal mitochondrial respiration.
Injection Protocol	Oligomycin → BAM15 → Rotenone plus Antimycin A → Monensin	Enables mitochondrial and glycolytic profiling from same cells and wells.
PER Data Correction	Apply H^+^/O_2_ ratio for mitochondrial contributions.	_mito_PER accounted for 24% of _total_PER under basal conditions.
Quality Control Thresholds	CV <20% for primary mitochondrial endpoints.	EasySep™ Direct achieves <10% CV at optimal conditions.

**Table 2:** Best practice recommendations for PBMC metabolic profiling based on experimental findings - key biomarker interpretations.

Biomarker	Key finding	Practical implication
Respiratory Control Ratio (RCR)	Sensitive indicator of processing time effects.	Significantly decreased with blood storage >48h - reliable early indicator.
Bioenergetic Health Index (BHI)	Limited sensitivity to early metabolic changes.	Only significant differences at 72h with SepMate™ - use with caution as sole biomarker.
Mitochondrial Toxicity Index (MTI)	Effective for mechanistic insights.	Revealed significant processing effects on mitochondrial oxidative capacity but not coupling control - useful for identifying and quantifying dysfunction type.

**Table 3:** Best practice recommendations for PBMC metabolic profiling based on experimental findings **-** troubleshooting common issues.

Problem	Likely cause	Recommended solution
High measurement variability (CV >30%)	Extended processing time or suboptimal seeding densities.	Use EasySep™ Direct for isolation, process blood within 24h, optimise cell density, consider using automated approaches for cell counting and pipetting.
RBC contamination visible	Density gradient isolation methods such as SepMate™ and/or delayed processing.	Switch to EasySep™ Direct for processing blood samples >24h.
Poor T-cell activation response	Blood storage >48 hours and or RBC contamination.	Process samples within 24h for activation studies.
Overestimated glycolytic rate	No correction for mitochondrial contributions to _total_PER. Or RBC contamination.	Apply H^+^/O_2_ correction factor in analysis. Consider switching to EasySep™ Direct for processing blood samples.
BHI shows no differences despite metabolic changes	BHI insensitivity to early dysfunction.	Use RCR or individual respiratory parameters as additional indicators.

## 4 Discussion

Results from the present study provide systematic evaluation of key factors influencing PBMC bioenergetic assessments using XF analysis. We confirmed a seeding density between 1x10^5^-3x10^5^ PBMCs per well for reproducible OCR and ECAR measurements, which is consistent with the literature (32–34). We also established a workflow with the sequential injection of oligomycin, BAM15 uncoupler, rotenone plus antimycin A and monensin to assess mitochondrial and glycolytic bioenergetic profiles of the same PBMCs (**Figure 2**). Our findings emphasise that blood processing time and isolation method significantly impact PBMC metabolism (**Figures 3**-**5**) and cell viability (**Supplementary Figure S2**). Prolonged blood storage (48–72 hours) at room temperature lowered mitochondrial function and increased glycolytic reliance, particularly in PBMCs isolated with the SepMate™ method, which were contaminated with red blood cells that confounded glycolytic measurements. Additionally, longer processing times led to impaired T cell activation capacity, highlighting the need for timely blood processing to preserve mitochondrial health. These insights have important implications for enhancing the accuracy, reproducibility, and reliability of metabolic assessments, particularly when profiling both mitochondrial and glycolytic flux of PBMCs using XF analysis.

Optimisation of seeding density is crucial for obtaining reliable measurements of OCR and ECAR when using XF analysis. PBMC seeding densities below 1×10^5^ cells per well produced OCR and ECAR values close to or below the detection limits of the XFe96 analyser. Conversely, seeding densities at the higher end (4×10^5^ cells per well) showed deviation from linearity between cell number and metabolic fluxes (**Figure 1**). The identification of an optimal PBMC seeding density range between 1×10^5^ and 3×10^5^ cells per well allowed for reliable detection of basal OCR and ECAR with capacity to measure metabolic activity. While this aligns with previous work (33, 34), our data emphasises that cell density must fall within the analyser sensitivity and higher cell densities do not always produce a linear response in OCR and ECAR. If rates fall outside this linear range, increased variation in specific mitochondrial respiratory parameters, including basal and maximal mitochondrial respiration (**Figure 1**) occurs. Therefore, this limits reproducibility and reliability of metabolic data when using PBMCs.

Extracellular acidification and consequent proton efflux from intact living cells is more complex than traditionally believed, arising from both glycolytic and respiratory sources (39). Respiring mitochondria contribute significantly to PER due to hydration of CO_2_ to carbonic acid (H_2_CO_3_), which rapidly dissociates into bicarbonate (HCO_3_) and a free proton (H^+^) (39). This acidification is particularly pronounced during active mitochondrial respiration, where increased metabolic flux amplifies CO_2_ generation and proton accumulation. Whilst theoretical H^+^/O_2_ ratios state a 1:1 stoichiometry (38), this is sensitive to the substrate being oxidised and inconsistent with XF microplates (37, 38). Recently, Desousa et al. empirically measured the H^+^/O_2_ ratio in over 20 cell types in XF microplates calculating an average of 0.38, which we used (**Supplementary Table S2**. Our study reveals that in PBMCs cultured with glucose, under basal conditions, PER stems 76% from glycolytic lactate production (_glyco_PER) and 24% from respiratory CO_2_ (_mito_PER) (**Figure 2B**). When mitochondrial ATP was inhibited with oligomycin, _mito_PER still accounted for 9% of _total_PER (**Figure 2B**). Whilst _total_PER increased by 25% after oligomycin (**Figure 2B**), following correction for _mito_PER, glycolytic lactate production (_glyco_PER) increased by 50%. Thus, compensatory glycolytic activity in PBMCs is significantly underestimated without correcting for _mito_PER (**Figure 2C**). This underscores the importance of correcting PER values for mitochondrial contributions to achieve accurate glycolytic measurements in PBMCs. Since we did not measure the H^+^/O_2_ ratio empirically in PBMCs, if this deviates significantly from 0.38, the calculated mitochondrial contributions to total PER could be over- or under-estimated (38), resulting in lower or higher _glyco_PER, respectively. However, employing the same calculation in all data sets does not alter the effect of blood processing time or isolation method on our downstream analyses.

In PBMCs, accurately assessing glycolytic capacity is crucial for understanding cellular metabolism. While previous studies have utilised monensin to enhance glycolytic activity in PBMCs (32, 33), our workflow uniquely combined monensin with the sequential injection of three key mitochondrial compounds: oligomycin, BAM15, rotenone, and antimycin A. We therefore examined the effect of these inhibitors on glycolytic activity. As anticipated, oligomycin significantly increased _glyco_PER, which was further increased but not significantly by rotenone plus antimycin A (**Figure 3B**). Notably, and consistent with previous findings in PBMCs and other cell types (32, 33, 40), monensin produced a further significant increase in _glyco_PER beyond rotenone and antimycin A alone (**Figure 2B**). Through strategic use of an ionophore to elevate glycolytic energy demand following compound-induced changes in mitochondrial respiration, we established a robust method for real-time metabolic profiling of PBMCs. Distinguishing this workflow from others, we are the first to incorporate the uncoupler BAM15 in such experiments for PBMCs. The data demonstrate that BAM15 maintains _glyco_PER integrity, addressing an important methodological consideration for real-time metabolic profiling (**Figure 2D**). These findings highlight the versatility of this injection strategy, enabling simultaneous monitoring of both oxidative and glycolytic ATP supply pathways from the same cell population, which is particularly valuable for bioenergetic studies using PBMCs. Our approach demonstrates that OCR and PER data, typically obtained through multiple injection strategies (32, 33, 40),, can be generated using a single combined protocol. Ultimately this will allow users to use less cells per experiment, lower consumable use, improve cost efficiencies and be more sustainable when using XF analysis for PBMC metabolic profiling.

To our knowledge, no previous study has systematically examined the direct effect of blood processing time on real-time cellular bioenergetics of PBMCs using XF analysis. Therefore, to expand on previous investigations (41–44), we investigated the direct impact of blood processing time and isolation method on the bioenergetic function of PBMCs. PBMCs isolated from blood samples stored at room temperature for 48 or 72 hours exhibited reduced mitochondrial respiration (**Figure 3**). This decline in mitochondrial function was accompanied by an increase in proton leak and consequent lower coupling efficiency (**Figure 3**), indicating a decrease in mitochondrial ATP supply. Moreover, extended processing time led to a decrease in compensatory glycolysis (**Figure 4**), consistent with the observed mitochondrial defects (**Figure 3**). A notable reduction in basal glycolytic activity and glycolytic capacity was also observed, but this was only in PBMCs isolated using EasySep™ Direct and not SepMate™. These metabolic changes likely reflect loss of metabolic fitness following prolonged blood storage, emphasising the importance of timely sample processing to preserve PBMC bioenergetics for downstream assessments. However, we cannot entirely exclude that changes in cell types comprising the PBMC fraction differ with increased processing time, which could contribute to metabolic parameters. This would likely be more prevalent at 72 hours when PBMC viability also significantly declines. Nonetheless, it is unlikely that viability alone is responsible for the metabolic decline, given that significant bioenergetics occur in PBMCs isolated from blood stored for 48 hours prior to any significant viability changes (**Supplementary Figure S2**). While future studies employing flow cytometric analysis would provide more definitive characterisation of cell type composition across processing times, our data confirm the importance of PBMC processing time for downstream functional analysis (41–44) and support that minimising processing delays to 24 hours or less is crucial for maintaining metabolic integrity and reducing preanalytical variability, which can significantly impact the reliability and interpretability of PBMC bioenergetics.

Notably, the method of PBMC isolation affected glycolytic outcomes. While both SepMate™ and EasySep™ Direct protocols yielded PBMCs with similar altered mitochondrial function after 48 and 72 hours of blood storage, extended blood processing time (48 and 72 hours) showed red blood cell contamination in PBMCs isolated using SepMate™ (**Supplementary Figure S4A**), which likely contributed to differences in glycolytic activity between the two methods (**Figure 4**). This may also explain the increased donor variability in metabolic parameters (**Figures 3**, **4** and **Supplementary Figure S1**). Our comprehensive presentation of individual data points through violin plots provides enhanced transparency regarding measurement variability and data distribution, revealing insights into different isolation method consistency. This approach demonstrates that EasySep™ Direct isolation yielded superior reproducibility across inter-donor repeats and processing times, with coefficient of variation values consistently below 25% for most parameters (**Supplementary Figure S1**). In contrast, SepMate™ isolation exhibited increased variability, particularly for glycolytic measurements at extended processing times, where some parameters exceeded 30% CV at 72 hours (**Supplementary Figure S1**). Specifically, PBMCs isolated using SepMate™ exhibited higher glycolytic rates compared to EasySep™ Direct isolated cells, particularly under conditions of increased blood processing time (**Figure 4**). This suggests that red blood cells, which rely predominantly on glycolysis, confound glycolytic measurements in PBMC isolations. Furthermore, red cell lysis using ammonium chloride (AC) had a detrimental impact on PBMC metabolism (**Supplementary Figure S4**). Suggesting that use of AC as a red cell lysis agent is not suitable for PBMC bioenergetic readouts. Additionally, the metabolic characteristics typical of T-cell activation were obscured in PBMCs isolated with red cell contamination. The visualisation of complete data distributions, rather than summary statistics alone, allows for better assessment of method reliability and facilitates more informed interpretation of bioenergetic parameters in clinical and research applications, particularly when evaluating different isolation protocols for extended processing conditions.

Consistent with observations on mitochondrial respiration, mitochondrial ATP supply in PBMCs isolated from blood processed after 48 and 72 hours was decreased (**Figure 5**). The decrease in mitochondrial ATP supply aligns with a decrease in mitochondrial function and an increase in glycolytic dependence, especially in cells isolated by SepMate™ (**Figures 3** and **4**). The distinction in glycolytic ATP supply between isolation method indicates that blood processing time affects mitochondrial and glycolytic pathways differentially by isolation method. Moreover, the glycolytic index of PBMCs (i.e., glycolytic ATP as a proportion of total ATP) revealed that prolonged blood processing time shifts PBMCs toward a more glycolytic phenotype, particularly in cells isolated using SepMate™ (**Figure 5**). Notably, when mitochondrial ATP was expressed relative to glycolytic ATP, it underscored the glycolytic shift in PBMCs processed at later times, especially for cells isolated with SepMate™ (**Figure 5**). The increase in glycolytic reliance is likely metabolic compensation due to lower ATP supply from mitochondria. However, the apparent higher glycolytic index of PBMCs isolated with SepMate™ compared to EasySep™ Direct is likely attributed to red blood cell contamination contributing to enhanced glycolysis.

The differential sensitivity patterns observed between the BHI and more specific mitochondrial parameters (RCR and MTI) provide important insights into the clinical utility of these metrics in PBMC bioenergetic assessments. The BHI integrates reserve capacity, ATP-linked respiration, non-mitochondrial respiration, and proton leak to provide overall mitochondrial bioenergetic assessment (27). This composite metric has demonstrated clinical utility across diverse disease states including diabetic nephropathy, where reduced BHI values correlated with loss of metabolic flexibility (45), cardiovascular disease (20), cancer immunotherapy responses (46), metabolic disorders (22, 47), and Alzheimer’s disease (48). Our observation that BHI remained preserved until extended storage periods (72 hours) while RCR and MTI demonstrated changes at earlier time points (48 hours) reflects the idea that BHI identifies progressive deterioration before complete energetic failure occurs (27). This suggests that while individual parameters are more sensitive to acute mitochondrial stress, the integrated BHI measure detects sustained metabolic perturbations with greater clinical relevance. However, the inconsistent BHI response - being significantly affected only in PBMCs isolated from 72-hr rested blood using SepMate™ - raises concerns about its reliability in the context of pre-analytical variability and highlights the need for careful consideration of methodological factors in clinical implementation.

These findings have significant translational implications. Studies employing BHI as a primary endpoint may have different tolerance for processing delays compared to those using individual parameters, affecting multi-center trials and biobanking protocols where delays are inevitable. The MTI analysis revealed that prolonged processing primarily affects mitochondrial oxidative capacity rather than coupling efficiency (**Figures 6C, D**), providing a pragmatic tool for assessing respiratory parameters in translational settings. Future directions should establish processing time thresholds for different BHI clinical applications and validate differential sensitivity patterns across disease states. Integration with other biomarkers such as mitochondrial DNA content may provide more comprehensive mitochondrial health evaluations as blood-based bioenergetics moves toward routine clinical implementation in personalised medicine (49–53).

Importantly, prolonged blood processing time (>24 hours) not only affects mitochondrial and glycolytic function but also impairs PBMC viability (**Supplementary Figure S2**) and energy production capacity during T-cell activation (**Figure 7**). This decrease in activation capacity, particularly in SepMate™-isolated PBMCs (**Figure 7G**), highlights the detrimental effects of delayed blood processing on immune cell functionality and metabolic reprogramming. While we cannot exclude the possibility that differential cell type composition within PBMC fractions may vary between isolation methods and processing times, particularly at 72 hours when viability is compromised, our findings emphasize that optimal PBMC isolation protocols should minimise processing time and carefully consider isolation method to preserve mitochondrial function and metabolic integrity.

## 5 Conclusion

This study provides systematic evaluation of critical parameters for accurate metabolic profiling of PBMCs using XF analysis. Our findings demonstrate that blood processing delays beyond 24 hours significantly impair mitochondrial function, glycolytic capacity, and T-cell activation. The EasySep™ Direct isolation method proved superior for blood stored beyond 24 hours, as SepMate™ resulted in red blood cell contamination that confounded glycolytic measurements and increased inter-donor variability. We developed a comprehensive injection strategy combining oligomycin, BAM15, rotenone/antimycin A, and monensin to simultaneously assess mitochondrial and glycolytic pathways. Importantly, our analysis revealed differential sensitivity patterns between integrated bioenergetic metrics (BHI) and individual mitochondrial parameters (RCR, MTI). The BHI showed selective preservation until extended processing delays, while individual parameters demonstrated earlier sensitivity to pre-analytical stress. This has significant implications for translational research, as studies employing integrated biomarkers like BHI may tolerate longer processing delays than those using individual mitochondrial parameters, affecting clinical trial design and biobanking protocols. Our methodological considerations enhance the reliability of PBMC-based bioenergetic assessments for clinical applications. Future studies should validate these parameters in disease-specific contexts to strengthen the translational potential of PBMC metabolic profiling as biomarkers for inflammatory and metabolic disorders. Standardised processing protocols within 24 hours remain essential for maintaining metabolic integrity and research reproducibility, with careful metric selection based on specific clinical or research applications.

## Data Availability

The raw data supporting the conclusions of this article will be made available by the authors, without undue reservation.
