# Supplementary material for: Comprehensive real-time metabolic profiling of peripheral blood mononuclear cells reveals important methodological considerations for immunometabolism research
Source: Front Immunol. 2025 Oct 29;16:1676550. doi: 10.3389/fimmu.2025.1676550 (PMC12605198; doi:10.3389/fimmu.2025.1676550)
Supplement: Supplementary file 1 [file Supplementaryfile1.docx]

**Supplementary material**

**Table 1.0.** Descriptive statistics of white blood cell counts from haematology analysis of blood samples taken across multiple days prior to PBMC isolation. Date are presented as means ± standard deviation from 3 different blood sampling occasions. White blood cells (WBC), large immature cells (LIC). Unit of measurement is 10^6^ cells/mL for each cell type.

|  | **Day 1 (72 hr)** | **Day 2 (48 hr)** | **Day 3 (24 hr)** | **Day 4 (Fresh)** |
| --- | --- | --- | --- | --- |
| **Total WBC** | 5.58 ± 0.31 | 5.90 ± 0.46 | 6.24 ± 0.76 | 6.48 ± 1.65 |
| **Neutrophils** | 3.06 ± 0.31 | 3.15 ± 0.34 | 3.48 ± 0.70 | 3.94 ± 1.50 |
| **Lymphocytes** | 1.85 ± 0.01 | 1.98 ± 0.04 | 1.97 ± 0.04 | 1.80 ± 0.08 |
| **Monocytes** | 0.42 ± 0.05 | 0.47 ± 0.06 | 0.49 ± 0.02 | 0.50 ± 0.11 |
| **Eosinophils** | 0.16 ± 0.03 | 0.18 ± 0.01 | 0.18 ± 0.01 | 0.15 ± 0.04 |
| **Basophils** | 0.07 ± 0.01 | 0.09 ± 0.00 | 0.09 ± 0.00 | 0.08 ± 0.01 |
| **LIC** | 0.03 ± 0.00 | 0.03 ± 0.01 | 0.04 ± 0.01 | 0.03 ± 0.01 |

|  | **Day 1 (72 hr)** | **Day 2 (48 hr)** | **Day 3 (24 hr)** | **Day 4 (Fresh)** |
| --- | --- | --- | --- | --- |
| **Total WBC** | 5.58 ± 0.31 | 5.90 ± 0.46 | 6.24 ± 0.76 | 6.48 ± 1.65 |
| **Neutrophils** | 3.06 ± 0.31 | 3.15 ± 0.34 | 3.48 ± 0.70 | 3.94 ± 1.50 |
| **Lymphocytes** | 1.85 ± 0.01 | 1.98 ± 0.04 | 1.97 ± 0.04 | 1.80 ± 0.08 |
| **Monocytes** | 0.42 ± 0.05 | 0.47 ± 0.06 | 0.49 ± 0.02 | 0.50 ± 0.11 |
| **Eosinophils** | 0.16 ± 0.03 | 0.18 ± 0.01 | 0.18 ± 0.01 | 0.15 ± 0.04 |
| **Basophils** | 0.07 ± 0.01 | 0.09 ± 0.00 | 0.09 ± 0.00 | 0.08 ± 0.01 |
| **LIC** | 0.03 ± 0.00 | 0.03 ± 0.01 | 0.04 ± 0.01 | 0.03 ± 0.01 |

**Table 2.0.** Formula for calculating glycolytic PER, ATP supply flux and respiratory control metrics including RCR, BHI and MTI.

| **Respiratory Parameter** | **Formula** |
| --- | --- |
| Basal glycolytic PER (glycoPER) | **Basal glycoPER** = (H^+^Total - 0.38 × OCR_Mito_)  where:  H^+^Total = total H^+^ production rates measured by the instrument  0.38 = average H^+^:O_2_ value (empirically determined across 21 cell types, ref 38)  OCR_Mito_ = basal mitochondrial oxygen consumption rate (corrected for non-mitochondrial oxygen consumption rate) |
| Oligo glycolytic PER (glycoPER) | **Oligo glycoPER** = (H^+^Total - 0.38 × OCR_oli_)  where:  H^+^Total = total H^+^ production rates measured by the instrument  0.38 = average H^+^:O_2_ value (empirically determined across 21 cell types, ref 38)  OCR_oli_ = mitochondrial oxygen consumption rate in the presence of oligomycin (corrected for non-mitochondrial oxygen consumption rate) |
| BAM15 glycolytic PER (glycoPER) | **BAM15 glycoPER** = (H^+^Total - 0.38 × OCR_bam15_)  where:  H^+^Total = total H^+^ production rates measured by the instrument  0.38 = average H^+^:O_2_ value (empirically determined across 21 cell types, ref 38)  OCR_bam15_ = mitochondrial oxygen consumption rate in the presence of BAM15 (corrected for non-mitochondrial oxygen consumption rate) |
| Basal mitochondrial PER | **mitoPER** = (0.38 × OCR_mito_)  where:  0.38 = average H^+^:O_2_ value (empirically determined across 21 cell types, ref 38)  OCR_mito_ = basal mitochondrial oxygen consumption rate (corrected for non-mitochondrial oxygen consumption rate) |
| Oligo mitochondrial PER | **mitoPER** = (0.38 × OCR_oli_)  where:  0.38 = average H^+^:O_2_ value (empirically determined across 21 cell types, ref 38)  OCR_oli_ = mitochondrial oxygen consumption rate in presence of oligomycin (corrected for non-mitochondrial oxygen consumption rate) |
| BAM15 mitochondrial PER | **mitoPER** = (0.38 × OCR_bam15_)  where:  0.38 = average H^+^:O_2_ value (empirically determined across 21 cell types, ref 38)  OCR_bam15_ = mitochondrial oxygen consumption rate in presence of BAM15 (corrected for non-mitochondrial oxygen consumption rate) |
| Basal oxidative phosphorylation ATP supply rate (ATPmito) | ATPmito = 5.45 × (OCR_ATP_ + 0.1 × OCR_Leak_)  Where:  5.45 = P:O_2_ (moles of ADP phosphorylated per O_2_ consumed)  OCRATP = ATP-linked oxygen consumption rate (respiration sensitive to oligomycin)  OCRLeak = oxygen consumption rate associated with proton leak (respiration insensitive to oligomycin). |
| Basal glycolytic ATP supply rate (ATPglyc) | ATPglyc = 1.53 × (H^+^Total - 0.38 × OCR_Mito_)  where:  1.53 = average lactate:H+ value (empirically determined across 17 cell types, ref 38)  H^+^Total = total H^+^ production rates measured by the instrument.  0.38 = average H^+^:O_2_ value (empirically determined across 21 cell types, ref 38)  OCRMito = basal mitochondrial oxygen consumption rate (corrected for non-mitochondrial oxygen consumption) |
| Total ATP | **ATPTotal** = ATPmito + ATPGlyc |
| Respiratory control ratio (RCR) | (BAM15 OCR – RA OCR)  **RCR** =  (proton leak OCR – RA OCR) |
| Bioenergetic Health Index | (ATP coupled × spare capacity OCR)  **BHI** = log  (proton leak OCR × non-mitochondrial OCR)  **ATP coupled** = ((Basal OCR-Oligo OCR)/BAM15 OCR)) x100  **Spare capacity** = ((BAM15 OCR-Basal OCR)/BAM15 OCR)) x100  **Non-mitochondrial** = (RA OCR/BAM15 OCR) x100  **Proton Leak** = 100-(ATP coupled+Spare capacity+non-mitochondrial) |
| Mitochondrial Toxicity Index for inhibition (MTI inhibitor) | (Max OCR_test_ – Max OCR_control_)  **MTI inhibitor** =  (Max OCR_control_ – Max OCR_RA_)  Where:  Max OCR_control_ = OCR after BAM15 addition from freshly isolated PBMCs  Max OCR_test_ = OCR after Bam15 addition from PBMCs isolated at different processing times (24hr, 48hr or 72hr)  Max OCR_RA_ = OCR after BAM15 and rotenone plus antimycin a addition from freshly isolated PBMCs |
| Mitochondrial Toxicity Index for uncoupling (MTI uncoupler) | (Oligo OCR_test_ – Oligo OCR_control_)  **MTI uncoupler** =  (Max OCR_control_ – Oligo OCR_control_)  Where:  Max OCR_control_ = OCR after BAM 15 addition from freshly isolated PBMCs  Oligo OCR_test_ = OCR after oligomycin addition from PBMCs isolated at different processing times (24hr, 48hr or 72hr)  Oligo OCR_control_ = OCR after oligomycin addition from freshly isolated PBMCs |

**Table 3.0.** Summary of basal mitochondrial respiration and maximum mitochondrial respiratory capacity across different cell seeding densities comparing EasySep^TM^ and SepMate^TM^ isolation methods. Data represent mean ± standard deviation from three independent experiments (n=3). Basal and Max values are expressed in pmol O₂/min/well. Coefficient of variation (CV%) was calculated as (standard deviation/mean) × 100. Quality assessment was based on CV% thresholds: Excellent (<10%), Good (10-20%), Fair (20-30%), and Poor (>30%). Seeding densities are expressed as thousands of cells per well (50k = 50,000 cells/well, etc.). Overall assessment summarises the combined performance of both respiratory parameters for each method-density combination.

| **Method** | **Seeding Density** | **Basal Mean±SD** | **Basal CV %** | **Basal Quality** | **Max Mean±SD** | **Max CV%** | **Max Quality** |
| --- | --- | --- | --- | --- | --- | --- | --- |
| **EasySep^TM^** | 50K | 10.0 ± 0.24 | 2.4 | **Excellent** | 32.4 ± 10.1 | 31.1 | **Poor** |
|  | 100K | 20.7 ± 1.1 | 5.3 | **Excellent** | 79.2 ± 3.4 | 4.3 | **Excellent** |
|  | 200K | 28.3 ± 5.3 | 18.6 | **Good** | 126.4 ± 18.9 | 14.9 | **Good** |
|  | 400K | 40.5 ± 4.0 | 9.8 | **Excellent** | 164.9 ± 17.3 | 10.5 | **Good** |
| **SepMate^TM^** | 50K | 15.6 ± 9.3 | 60 | **Poor** | 62.5 ± 50.0 | 80 | **Poor** |
|  | 100K | 18.3 ± 3.0 | 16.2 | **Good** | 68.7 ± 15.2 | 22.2 | **Fair** |
|  | 200K | 20.5 ± 3.7 | 18.2 | **Good** | 92.0 ± 31.8 | 34.6 | **Poor** |
|  | 400K | 37.7 ± 7.2 | 19.2 | **Good** | 158.4 ± 59.7 | 37.7 | **Poor** |

.

**Supplementary Figure 1.** Coefficients of variation (CV in %) from metabolic parameters established from XF analysis of PBMCs isolated after different processing times (0h, 24h, 48h or 72h) from EasySep Direct^TM^ (**A**) or SepMate^TM^ (**B**) isolation method. CV’s are presented from 8-12 technical replicates from 3 separate blood donations from an individual donor.

**Supplementary Figure 2.** Viability of PBMCs isolated after different processing times (0h, 24h, 48h or 72h) from EasySep Direct^TM^ (**blue**) or SepMate^TM^ (**red**) bars. Data are presented as mean cell viabilities ± SEM from 3 separate isolations from blood of an individual donor.

**
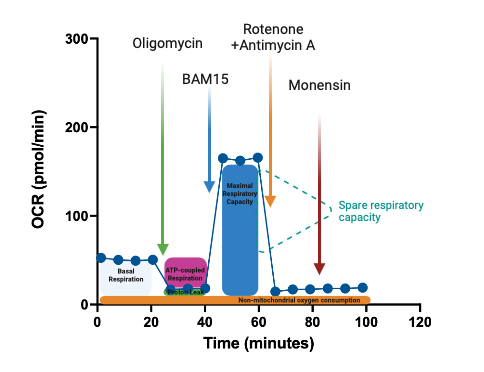
 A.**


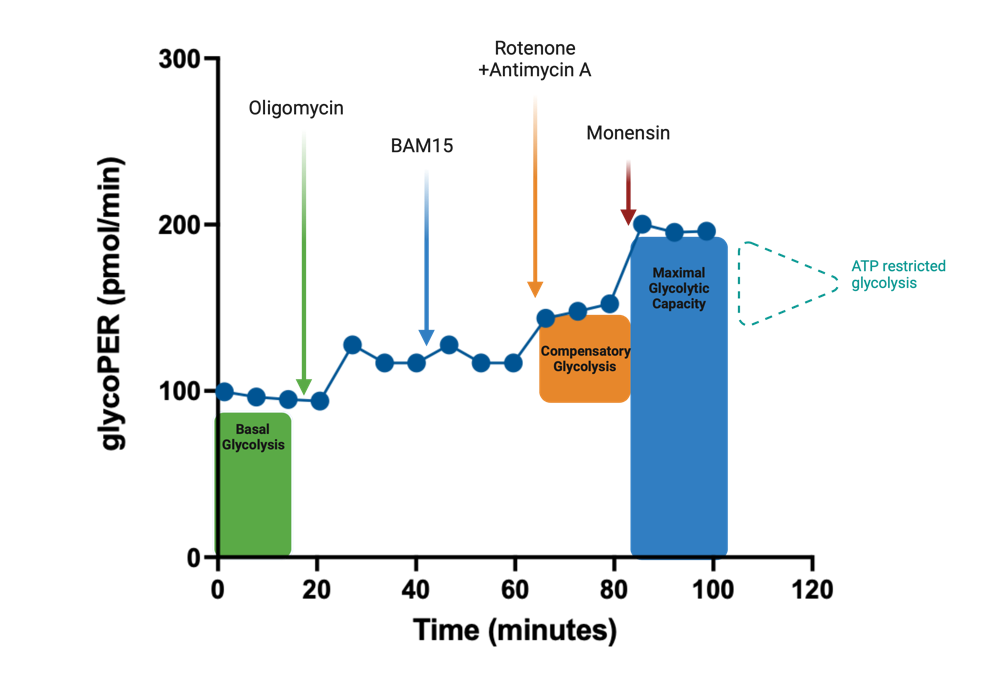
**B.**

**Supplementary Figure 3. Examples of real-time metabolic profiling assay: Oxygen consumption rate (OCR) and glycolytic proton efflux rate (glycoPER).** (A) Kinetic trace of oxygen consumption rate (OCR) following the sequential injection of metabolic inhibitors: oligomycin (to assess ATP-coupled respiration and proton leak), BAM15 (to measure maximal respiratory capacity), rotenone plus antimycin A (to measure non-mitochondrial respiration). The trace is annotated to highlight key phases of the mitochondrial respiratory profile: basal respiration (prior to any injections), ATP-coupled and proton leak (following oligomycin injection, maximal respiratory capacity (post-BAM15), and non-mitochondrial respiration (post-rotenone + antimycin A). Spar respiratory capacity is calculated by subtracting basal OCR from maximal OCR. (B) Kinetic trace of glycolytic proton efflux rate (glycoPER). Basal glycolytic activity is measured prior to any injections. Compensatory glycolysis is assessed following the injection of rotenone and antimycin A, which inhibits mitochondrial respiration and shifts cellular metabolism toward glycolysis. Maximal glycolytic capacity is determined after monensin injection, wherby activation of Na+/K+ ATPase increaes energy demand further. ATP-restricted glycolysis is calculated by subtracting compensatory glycolysis from maximal glycolysis, providing a measure of glycolytic energy supply not dependent on mitochondrial ATP compensation.


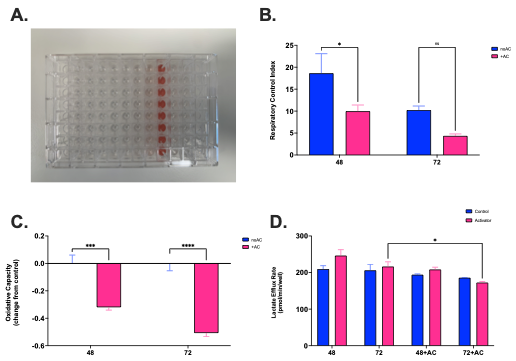


**Supplementary Figure 4.** **Assessment of PBMC isolation and metabolic responses post-separation using SepMate™ ± ammonium chloride treatment.** (A) Image of a 96-well microplate showing PBMCs seeded after isolation, with visible red blood cell contamination resulting from SepMate™-based isolation. (B) Respiratory control index of PBMCs isolated from blood processed after 48 hr or 72 hr using SepMate™ with and without ammonium chloride (AC) treatment. (C) Oxidative capacity of PBMCs, assessed via mitochondrial toxicity index (MTI), from blood processed after 48 hr or 72 hr using SepMate™ with and without ammonium chloride (AC). (D) Lactate efflux rate of PBMCs isolated from blood processed after 48 hr and 72 hr using SepMate™ with and without ammonium chloride (AC), and treated with or without CD3/CD28 immunocult T-cell activator. Data are presented as means ± SEM from 4 technical replicates. Statistical differences were assessed by Two-Way ANOVA using Fisher’s LSD post hoc analysis (*P<0.05, ***P<0.001, ****P<0.0001).
